# Mortality predictors in septic HIV patients in the ICU

**DOI:** 10.1186/cc12406

**Published:** 2013-03-19

**Authors:** P Vidal-Cortés, P Lameiro-Flores, A Aller-Fernández, M Mourelo-Fariña, P Fernández-Ugidos, R Gómez-López, M Alves-Pérez, E Rodríguez-García

**Affiliations:** 1 CHU Ourense, Spain; 2 CHU A Coruña, Spain

## Introduction

Sepsis in HIV patients carries a high mortality. Our purpose is to identify mortality predictors in this population.

## Methods

A retrospective study of HIV patients admitted to our ICU between January 2005 and December 2009. We identify septic patients and analyzed demographic factors, etiology, organ failure and outcome. We used logistic regression to calculate the relative mortality risk for each qualitative variable and the beta (β) coefficient for quantitative variables.

## Results

Sixty-two septic patients were admitted to our ICU during the study period (71% men, mean age: 40.59 ± 8.12). A total of 56.5% came from the ED, mean hospital stay before ICU admission: 6.14 ± 10.96 days. A total of 66.1% had a history of intravenous drug use, other comorbidities: COPD (9.7%), cirrhosis (8.1%), solid or hematologic malignancy (12.9%). A total of 40.3% were under HAART. Mean CD4 count at admission: 219.62 ± 353.93 cells/mm^3^. Mean viral load: 4.57 ± 3.25 log. Mean albumin levels: 2.3 ± 0.53 g/dl. APACHE II: 21.98 ± 7.97. The lung was the most frequent focus of infection (65.6%) followed by the CNS. The most common pathogen was *S. pneumoniae *(28.8%), followed by *P. jirovecii *(13.6%). In total, 62.9% needed vasopressors, 79% mechanical ventilation and 19.4% renal replacement. Mean ICU and hospital length of stay: 10.4 ± 10.52 and 34.76 ± 29.64 days. ICU mortality: 33.9%; hospital mortality: 41.9%. ICU mortality increases for each days of hospital stay before ICU admission (β = 1.062, 95% CI = 1.009 to 1.118, *P *= 0.022), each point of: cardiac frequency at admission (β = 1.059, 95% CI = 1.024 to 1.096, P = 0.001), FiO_2 _(β = 1.034, 95% CI = 1.011 to 1.057, P = 0.003), pCO_2 _(per mmHg) (β = 1.052, 95% CI = 1.007 to 1.098, *P *= 0.023); and decreases for each point of: albumin (g/dl) (β = -0.307, 95% CI = 0.095 to 0.986, *P *= 0.047), mean arterial pressure (mmHg) (β = 0.951, 95% CI = 0.904 to 0.999, *P *= 0.048), pH (β = 0.002, 95% CI = 0.0 to 0.172, *P *= 0.006), first ICU 24-hour diuresis (ml) (β = 0.999, 95% CI = 0.998 to 1.000, *P *= 0.004) and base excess (β = 0.912, 95% CI = 0.836 to 0.995, *P *= 0.039). Mortality increases threefold (β = 3.134, 95% CI = 1.051 to 9.345, *P *= 0.04) if patients do not come from the ED, almost 10-fold if the patients need vasopressors for more than 24 hours (β = 9.975, 95% CI = 2.054 to 48.451, *P *= 0.004) and fivefold if patients need renal replacement (β = 5.692, 95% CI = 1.466 to 22.099, *P *= 0.012).

## Conclusion

Neither immune status-related variables nor comorbidity or infection focus are mortality predictors. Poor nutritional status, delayed ICU admission, shock or renal failure increase the ICU relative mortality risk. Tachycardia, hypotension, hypercapnia, acidosis, and oliguria in the first ICU 24 hours increase significantly ICU mortality. Mechanical ventilation is not a mortality predictor.

